# Recurrence of nasal type NK/T cell lymphoma presenting as neurolymphomatosis on ^18^F-FDG PET/CT: A case report and literature review

**DOI:** 10.1097/MD.0000000000018640

**Published:** 2020-01-03

**Authors:** Qingqing Pan, Yaping Luo

**Affiliations:** a Department of Nuclear Medicine, Chinese Academy of Medical Sciences and Peking Union Medical College Hospital; b Beijing Key Laboratory of Molecular Targeted Diagnosis and Therapy in Nuclear Medicine; Beijing, PR China.

**Keywords:** FDG, neurolymphomatosis, NK/T cell lymphoma, PET/CT

## Abstract

**Introduction:**

NK/T cell lymphomas seldom involve the peripheral nervous system. We report a case of recurrent nasal type NK/T cell lymphoma presenting as neurolymphomatosis and its manifestation on ^18^F-FDG PET/CT.

**Patient concerns:**

A 55-year old man presented with a mass in the right nasal cavity was diagnosed with extranodal NK/T cell lymphoma, nasal type. ^18^F-FDG PET/CT showed intense FDG uptake within the mass. After radiotherapy the nasal tumor was completely relieved, but the patient experienced numbness and amyosthenia in the right upper extremity one week after completion of radiotherapy.

**Diagnosis:**

PET/CT showed intense FDG uptake in the brachial plexus, axillary, suprascapular and median nerves, suggestive of recurrence of lymphoma presenting as neurolymphomatosis.

**Interventions:**

After 1 cycle of chemotherapy, the follow-up PET/CT showed markedly reduced FDG uptake in the previous involved nerves, demonstrating a very good response of neurolymphomatosis to chemotherapy.

**Outcomes:**

The patient finally had a progression free survival of 8 months after completion of 4 cycles of chemotherapy and autologous stem cell transplantation.

**Lessons:**

As neurolymphomatosis is a rare neurologic manifestation in recurrence of NK/T cell lymphoma, recognition of its presentation is important for prompt diagnosis and initiating treatment approach.

## 1 Introduction

Nervous system is occasionally affected in the setting of lymphoma, resulting from infiltration of neoplastic cells in nervous system or neurotoxicity secondary to chemotherapy, infections, immune-mediated, paraneoplastic or metabolic processes and nutritional deficiencies.^[1]^ Neurolymphomatosis (NL) is a term that encompasses direct infiltration by lymphoma cells in nerves. It most frequently involves peripheral nerves, followed by spinal nerve root, cranial nerve, and neural plexus.^[1,2]^ The most common type of lymphoma in NL is non-Hodgkin B-cell lymphoma, especially diffuse large B-cell lymphoma.^[2]^ Neuroimaging including magnetic resonance imaging (MRI) and ^18^FDG PET/CT has greatly contributed to the diagnostic work-up of NL.^[3,4]^ We herein report a case of nasal type NK/T cell lymphoma with recurrent disease presenting as NL.

## 2 Case presentation

Ethical approval was waived by the institutional review board of our hospital as this work is a retrospective case report. The patient has provided informed consent for publication of the case.

**Figure 1:**
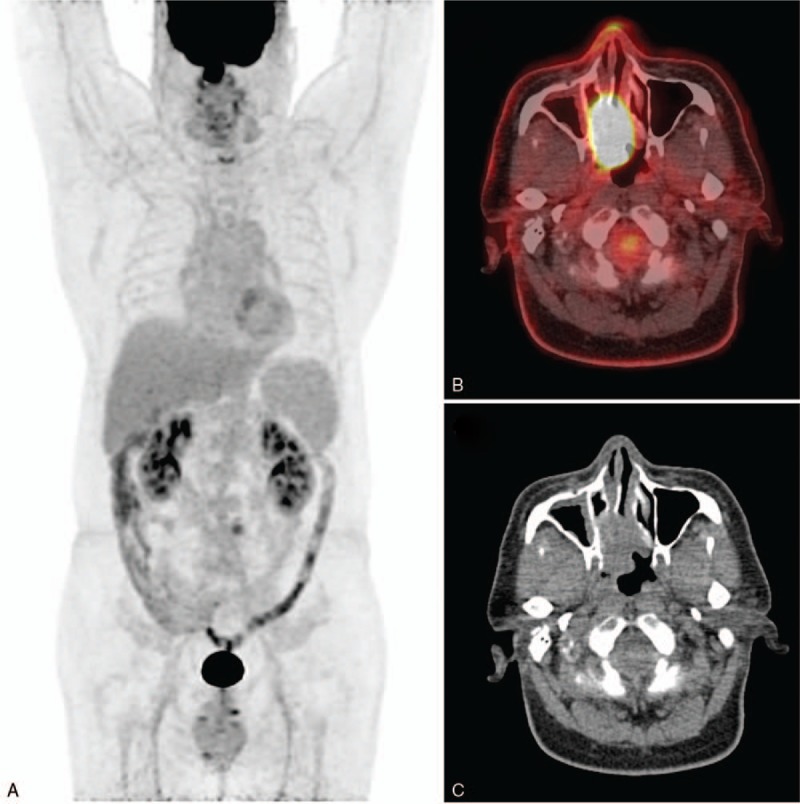
FDG PET/CT at baseline assessment. PET MIP (A), axial fusion image (B) and axial CT (C) showed a mass with intense FDG uptake in the right nasal cavity with size of 4.1×2.3 and SUVmax of 26.0.

**Figure 2:**
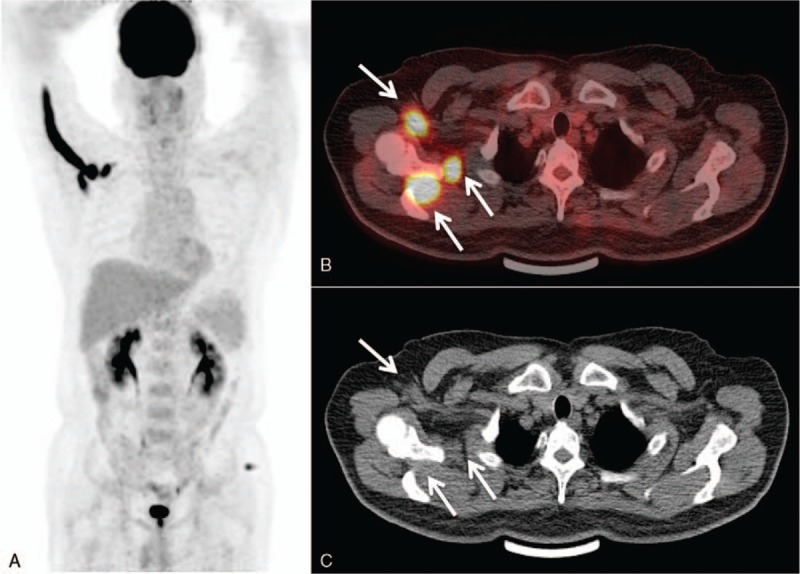
Follow-up PET/CT after radiotherapy. PET MIP (A), axial fusion image (B) and axial CT (C) revealed intense FDG uptake in the brachial plexus, axillary, suprascapular and median nerve with SUVmax of 19.9, suggesting recurrence of lymphoma.

**Figure 3:**
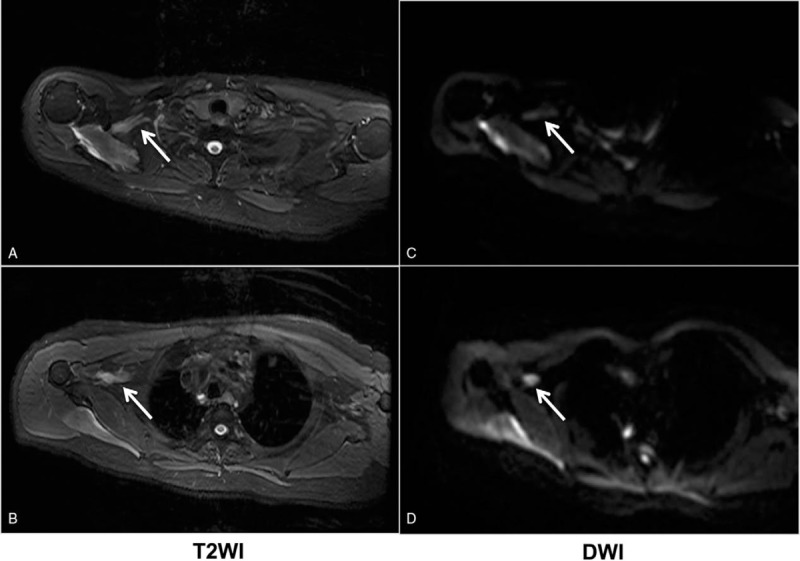
MR images showed the right brachial plexus and axillary nerves had hypersignal intensity in T2WI (A-B); the involved nerves were also hyperintense in DWI (C-D).

A previously healthy 55-year old man presented with a runny nose for 6 months. MRI images revealed a mass in the right nasal cavity, which was subsequently diagnosed with biopsy as extranodal NK/T cell lymphoma, nasal type. Serum Epstein-Barr virus (EBV)-DNA was 2000 copies/ml. ^18^F-FDG PET/CT was then performed for staging of the lymphoma. PET/CT images (Biograph 64 mCT, Siemens, Germany) were acquired 1 hour after intravenous injection of 387.6 MBq ^18^F-FDG, and the emission scan (2 min/bed, 2 iterations, 21 subsets) was obtained from the tip of the skull to the mid-thigh. ^18^F-FDG PET/CT (Fig. 1) showed a mass with intense FDG uptake in the right nasal cavity. The mass was measured 4.1 × 2.3 cm in size, and the SUVmax was 26.0. No other hypermetabolic lesions were noted. The patient then received 1 cycle of radiotherapy (total dose 110 Gy), after which a follow-up MRI showed complete response of the tumor. About one week after completion of radiotherapy, the patient had a progressive numbness, amyosthenia and pain in the right upper extremity. The brain and cervical spine MRI showed lacunar infarction and intervertebral disc bulge in C3/4-C6/7, but it could not explain the patient's symptoms. Electromyogram indicated right median nerve injury. Serum EBV-DNA increased to 7800 copies/ml. ^18^F-FDG PET/CT (407 MBq, uptake time 67 minutes) was then repeated for the underlying recurrence of lymphoma. In the follow-up PET/CT (Fig. 2), intense FDG uptake was noted in the brachial plexus, axillary, suprascapular, and median nerve (SUVmax 19.9), suggestive of recurrence of lymphoma in the above nerves. The nasal cavity and paranasal sinus were normal. Consistent with the findings in PET/CT, MRI (Fig. 3) showed hypersignal intensity in the involved nerves in T2WI and DWI. Considering the difficulty to perform biopsy in these nerves and the potential neurological sequelae after biopsy, chemotherapy was directly initiated. After one cycle of SMILE chemotherapy (methotrexate, dexamethasone, ifosfamide, VP16, and pegaspargase), his pain and numbness in the right upper extremity was somewhat relieved. The follow-up ^18^F-FDG PET/CT showed marked reduction of FDG uptake in the previous involved nerves (SUVmax 3.7), demonstrating a very good response to chemotherapy. Then the patient continued to receive another 3 cycles of SMILE chemotherapy and subsequently had autologous stem cell transplantation. After completion of the above treatments, serum EBV-DNA returned to normal and another follow-up PET/CT showed no evidence of disease. Eight months later, the patient again experienced progressive and disseminated disease involving multiple lymph nodes, pancreas and bone marrow. The patient was then recruited in the clinical trial of PD-1 therapy.

## 3 Discussion

NL, a rare neurologic manifestation of lymphoma, could be primary involvement of lymphoma cells exclusively in peripheral nervous system, or secondary to systemic involvement from other tissue and organs. It may occur at initial diagnosis of lymphoma or as a relapse or progression of a previously treated disease.^[4,5]^ Clinical manifestation of NL is variable due to different site and extent of disease. Common complaints of patients with NL include painful peripheral neuropathy (present in 76% of patients),^[2]^ loss of sensation or motor function (usually a mixed sensorimotor neuropathy),^[4,6]^ and cranial neuropathy.^[5,7]^ In the current case, the patient had both painful peripheral neuropathy and a mixed sensorimotor neuropathy in the upper extremity, which was the only clinical presentation of recurrent disease. This early recognition initiated the diagnostic approach of PET/CT and the subsequent treatment with good response to therapy. Thus if a patient with a known history of lymphoma presents with neurologic manifestation of peripheral nervous system, NL should be considered.

According to a retrospective analysis of 50 cases of NL, the most common type of lymphoma in NL was diffuse large B-cell lymphoma (75.5%), followed by follicular lymphoma (9%), peripheral T-cell lymphoma (4%) and mantle cell lymphoma (1%).^[2]^ NK/T cell lymphoma involving central or peripheral nerve system is exceedingly rare with only sporadic cases reported in English literature.^[8–20]^ In previous reported cases, the location of NL with NK/T cell lymphoma included brachial plexus, median nerve, ulnar nerve, tibial nerve, peroneal nerve, sural nerve, and lumbar nerve roots.^[8–13]^ In the current case, brachial plexus, axillary, suprascapular and median nerves were involved, which was consistent with the predilection of localization in NL with NK/T cell lymphoma.

The diagnosis of NL is complicated and challenging. Histopathology of nerve biopsy demonstrating neural infiltration by malignant cells is the gold standard for diagnosis. The case series reported by The International Primary Central Nervous System Lymphoma Collaborative Group showed a positive rate of 88.5% for nerve biopsy; however, 11.5% of patients were false negative.^[2]^ In some cases, histopathological diagnosis of NL could not be established until autopsy.^[5]^ Besides the difficulty to obtain adequate tissue in nerve biopsy, complications as trauma, bleeding, and neurologic sequela resulting from nerve biopsy are also important issues to consider. Therefore, neuroimaging has become increasingly important in the diagnostic work-up of NL. MRI and ^18^F-FDG PET/CT are most commonly used in these circumstances. MRI findings of NL usually include diffuse or nodular thickening of nerves, hypointensity on T1WI and hyperintensity on T2WI, and abnormal enhancement of the affected neural structure.^[2,21–23]^ NL on ^18^F-FDG PET usually presented with linear and nodular hypermetabolic pattern with or without prominent CT abnormalities. The FDG avidity in NL lesions was moderate to high with SUVmax ranging from 2.3 to 13.7.^[3,4,24–26]^ According to previous literature, the diagnostic yield of MRI in NL was 59% to 77%, while that of FDG-PET was 84% to 100%, suggesting a higher sensitivity of FDG-PET than MRI in diagnosing NL.^[2–5,24]^ In our case, the recurrence of lymphoma was first detected by FDG PET/CT presenting as FDG-avid abnormalities in multiple nerves. It again emphasized the importance of FDG PET/CT both with its high sensitivity in NL and full-extent assessment of the disease. Furthermore, FDG PET/CT may also help with differentiation between NL and paraneoplastic neuropathies in lymphoma,^[6]^ while the latter usually is a symmetrical demyelinating polyneuropathy presenting as Guillain-Barré syndrome or chronic inflammatory demyelinating polyneuropathy.^[21]^ Besides PET/CT, serum EBV-DNA was another important marker for surveillance in our case—it changed in parallel with disease progression and response to treatment. EBV is etiologically linked to a remarkably wide range of lymphoproliferative disease and malignant lymphomas especially with the Burkitt lymphoma, Hodgkin disease, diffuse large B cell lymphoma, extranodal NK/T cell lymphoma, aggressive NK leukemia, and primary central nervous system lymphoma.^[27–30]^ The quantification of EBV load is useful for prognostic assessment and evaluation of treatment responses in extranodal NK/T cell lymphoma and Hodgkin lymphoma.^[31–33]^

The median overall survival of patients with NL calculated from the diagnosis of NL was only 10 months,^[2,24]^ and there was no statistically significant difference in survivals between patients with primary and secondary NL.^[2]^ Another registry study from Baehring et al^[5]^ reported 52 patients with NL died within 2 weeks to 84 months after symptom onset. But to date, there is no evidence to state whether lymphoma patients with NL have inferior overall survival comparing to those without NL. Treatments for NL usually include systemic chemotherapy, intrathecal chemotherapy and radiotherapy.^[2]^ Systemic chemotherapy is used in cases with multiple sites of involvement, while radiotherapy has a limited role in this type of disease. But radiotherapy can be very effective in the control of chemo-refractory localized lymphomatous aggregates.^[2]^ Intrathecal chemotherapy may not be effective to treat all lesions of NL, as it usually involves nerves and nerve roots beyond the border of subarachnoid space.^[2,5]^ The response rate (complete and partial response) of NL to chemotherapy was reported to be 46% to 89%.^[2,24]^ Our patient had an early and very good response to treatment after one cycle of chemotherapy, but his disease finally progressed with a progression free survival of 8 months after NL was treated.

## 4 Conclusion

We have reported a case of nasal type NK/T cell lymphoma with recurrence presenting as NL. The role of FDG PET/CT in this case was highlighted in early diagnosis and prompt treatment against NL. Awareness of the clinical and imaging characteristics of this rare presentation of lymphoma may help with better theranostic work-up in clinical practice.

## Author contributions

**Conceptualization:** Yaping Luo.

**Data curation:** Qingqing Pan.

**Funding acquisition:** Qingqing Pan.

**Investigation:** Qingqing Pan.

**Supervision:** Yaping Luo.

**Writing – original draft:** Qingqing Pan.

**Writing – review & editing:** Yaping Luo.
